# Development of a high-resolution melt-based assay to rapidly detect the azole-resistant *Candida auris* isolates

**DOI:** 10.22034/CMM.2023.345114.1453

**Published:** 2023-09

**Authors:** Hamid Morovati, Hamid Badali, Mahdi Abastabar, Keyvan Pakshir, Kamiar Zomorodian, Bahram Ahmadi, Behrouz Naeimi, Sadegh Khodavaisy, Sanam Nami, Esmaeil Eghtedarnejad, Hossein Khodadadi

**Affiliations:** 1 Department of Parasitology and Mycology, School of Medicine, Shiraz University of Medical Sciences, Shiraz, Iran; 2 Department of Molecular Microbiology and Immunology, South Texas Center for Emerging Infectious Diseases, University of Texas at San Antonio, San Antonio, TX, USA; 3 Invasive Fungi Research Center, Communicable Diseases Research Institute, Mazandaran University of Medical Sciences, Sari, Iran; 4 Department of Medical Mycology, School of Medicine, Mazandaran University of Medical Sciences, Sari, Iran; 5 Basic Sciences in Infectious Diseases Research Center, Shiraz University of Medical Sciences, Shiraz, Iran; 6 Department of Medical Laboratory Sciences, Faculty of Paramedicine, Bushehr University of Medical Sciences, Bushehr, Iran; 7 Department of Medical Parasitology and Mycology, School of Public Health, Tehran University of Medical Sciences, Tehran, Iran; 8 Department of Parasitology and Mycology, Faculty of Medicine, Tabriz University of Medical Sciences, Tabriz, Iran

**Keywords:** Azole resistance, *Candida auris*, High-resolution melt curve analysis, Mutation screening, Real-time PCR

## Abstract

**Background and Purpose:**

*Candida auris* is a multidrug-resistant yeast that rapidly spreads, making it the leading Candidate for the next pandemic. One main leading cause of emerging resistant *C. auris* isolates is nonsynonymous mutations. This study aimed to detect the Y132F mutation, one of the most important azole resistance-associated mutations in the *ERG-11* gene of *C. auris*, by developing a reliable high-resolution melt (HRM)-based method.

**Materials and Methods:**

Five *C. auris* isolates from Iran, plus three control isolates from other Clades were used in the study. The antifungal susceptibility testing through micro broth dilution was performed to recheck their susceptibility to three azole antifungals, including fluconazole, itraconazole, and voriconazole. Moreover, the polymerase chain reaction (PCR) sequencing of the *ERG-11* gene was performed. Following the bioinformatic analysis and HRM-specific primer design, an HRM-based assay was developed and evaluated to detect *ERG-11* mutations.

**Results:**

The minimum inhibitory concentrations of fluconazole among Iranian *C. auris* isolates ranged from 8 to 64 μg/mL. The PCR-sequencing of the *ERG-11* gene and bioinformatic analyses revealed the mutation of Y132F, a substitution consequence of A to T on codon 395 in one fluconazole-resistant isolate (IFRC4050). The developed HRM assay successfully differentiated the targeted single nucleotide polymorphism between mutant and wild types (temperature [Tm]: 81.79 ℃ - cycle threshold [CT]: 20.06 for suspected isolate). For both mutant and non-mutant isolates, the mean Tm range was 81.79-82.39 °C and the mean CT value was 20.06-22.93. These results were completely in accordance with the findings of DNA sequencing.

**Conclusion:**

The fast-track HRM-based method successfully detected one of the most common mechanisms of resistance in the *ERG-11* gene of *C. auris* within 3 h. Finally, the development of more panels of HRM assays for the detection of all azole resistance mutations in *C. auris* *ERG-11* is recommended to expand the scope of the field and facilitate the elaboration of rapid and accurate methods of antifungal resistance assessment.

## Introduction

*Candida auris* is a multidrug-resistant (MDR) haploid yeast that causes severe nosocomial or non-nosocomial infections, especially in immunocompromised hosts, including broad-spectrum antibiotics and corticosteroids recipients, patients undergoing hemodialysis and intensive chemotherapy, and those with severe malignancies, diabetes, and extended intensive care unit stays (*e.g.*, COVID-19 patients) [ 1 - 6 ]. *Candida auris* has different phylogenetic traits related to the geographical pattern of its distribution [ 7 - 10 ]. Therefore, *C. auris* isolates are divided into five geographical Clades, including South Asian (Clade I), East Asian (Clade II), South African (Clade III), South American (Clade IV), and Iranian (Clade V). Remarkably, these Clades are different by 100,000 to 200,0000 single nucleotide polymorphisms (SNPs) from each other (interclade) and some intraclade SNP variations between the isolates of each Clade [ 11 , 12 ]. Recently, an additional Clade VI, Indomalaya, was reported from Singapore [ 13 ].

One major issue that has raised global concerns is the emergence of MDR isolates of *C. auris* [ 3 , 14 - 16 ]. This causes several problems, such as raising treatment costs, requiring critical diagnosis, and prolonging antifungal therapies. Therefore, the detection of MDR isolates is highly crucial, and focusing on the development of novel, reliable, and time-efficient methods is a priority. Azoles are among the top antifungals used in the treatment of candidiasis [ 3 , 14 , 15 ], which inhibit the activity of 14-sterol demethylase encoded by the *lanosterol* *C14-demethylase* (*ERG-11*) gene. Ergosterol production is a key structural component of fungal cell membranes and is disrupted by these antifungals. Several *ERG-11*-related mechanisms lead to azole resistance, mainly nonsynonymous point mutations [ 3 , 14 , 15 ]. Therefore, further focus on this gene will pave the way for the detection of azole-resistant *C. auris* isolates. 

As mentioned before, one of the primary mechanisms underlying resistance to azole antifungal drugs is the presence of SNPs in the gene responsible for azole resistance. Among these SNPs, the Y132F substitution has gained attention due to its potential impact on the prevalence and clinical outcomes of *C. auris* infections. Even though the exact frequency of *ERG-11* Y132F in Clade V is still unknown, the frequency and prevalence of this mutation may vary among Clades. This is the most common mutation associated with azole resistance in Clades I and IV. However, each isolate from Clade III that was resistant to fluconazole had an *ERG-11* F126L mutation [ 17 , 18 ]. Therefore, tracking this mutation in Clad V could help to find out the interclade relations and the possibility of azole resistance. Therefore, the presence of this mutation in Clade V may indicate the presence of azole resistance and also help the Clade classification of the isolates.

High-resolution melt (HRM) analysis is a closed-tube, post-polymerase chain reaction (PCR) analysis method based on the measurement of the exact melting temperature of amplified DNA that is used for the identification of genetic variation in nucleic acid sequences [ 19 , 20 ]. This method is based on PCR product melting analysis and is enabled by the recent availability of improved double-stranded DNA (dsDNA)-binding dyes along with the next-generation real-time PCR instrumentation and analysis software. The HRM analysis can differentiate the tiny dissociation characteristics of dsDNAs during melting depending on the composition, length, GC content, or strand complementarity of the variant DNA sequences [ 21 ].

Recently, a study [ 18 ] developed a molecular beacon-based DNA melting curve assay for rapid detection of *ERG-11*-associated SNPs among *C. auris* isolates of Clades I-IV. However, they did not involve the fifth Clade isolates since Clade V was not validated at the time. Therefore, the authors of the present study decided to develop and evaluate a cost-effective, specific, and reliable HRM-based method for the detection of possible point mutation(s) in the *ERG-11* gene among several Clades of *C. auris*, including Clade V, which was specifically reported from Iran.

## Materials and Methods

### Isolates

Five isolates of *C. auris* reported from Iran were included in this study. These isolates were labeled Babol-1 (IFRC2087) [ 22 ], Babol-2 (IFRC4050) [ 23 ], Shiraz (TMML616), Tehran (CSF1020) [ 24 ], and Bushehr (MRL32) [ 25 ]. The matrix-associated laser desorption ionization-time of flight mass spectrometry, whole genome sequencing (WGS), and short tandem repeat (STR) typing for those isolates have been previously performed in our previous study [ 11 ]. The first three isolates belonged to Clade V, and the isolates CSF1020 and MRL32 belonged to Clade I. In addition, three standard *C. auris* strains from Clade II (CBS10913 and CBS12372) and Clade I (CBS14916) were included as controls (Table 1). All isolates were subcultured onto malt extract agar (MEA, Merck, Germany) on the day of receipt prior to further testing.

**Table 1:** Candida auris isolates used in the study and results of the antifungal susceptibility testing, bioinformatic analysis, Real-time polymerase chain reaction (PCR), and post-PCR high-resolution melt experiments

#	*C. auris* Clades	ID	Confirmed by	AFST (MIC: μg/mL)			SNP on codon 395 of the ERG-11 gene	Mean temperature (°C)	Mean CT value
				FLC	ITC	VRC			
1	Clade V	IFRC2087	MOLDI-TOF MS,	16	0.063	0.125	None	82.34	22.93
			STR, WGS						
2	Clade V	IFRC4050	MOLDI-TOF MS	64	0.016	1	395 A to T	81.79	20.06
			STR, WGS				Leads to amino acid substitution Y132F		
3	Clade V	TMML616	MOLDI-TOF MS	1	0.016	0.016	None	82.26	22.64
			STR, WGS						
4	Clade I	CSF1020	MOLDI-TOF MS	>64	0.125	0.25	None	82.15	21.41
			STR, WGS						
5	Clade I	MRL32	MOLDI-TOF MS	8	0.5	0.25	None	82.39	21.33
			STR, WGS						
6	Clade II	CBS10913	MOLDI-TOF MS	1	0.016	0.016	None	81.98	22.30
			STR, WGS						
7	Clade II	CBS12372	MOLDI-TOF MS	8	0.125	0.25	None	81.73	22.40
			STR, WGS						
8	Clade I	CBS14916	MOLDI-TOF MS	128	0.5	0.12	None	81.81	21.52
			STR, WGS						

AFST: antifungal susceptibility test, MIC: minimum inhibitory concentration, FLC: fluconazole, ITC: itraconazole, VRC: voriconazole, SNP: single nucleotide polymorphism, MOLDI-TOF MS: matrix-assisted laser desorption ionization–time-of-flight mass spectrometry, STR: short tandem repeat, WGS: whole genome sequencing, CT: cycle threshold

### Antifungal susceptibility testing

Antifungal susceptibility testing was performed for fluconazole, itraconazole, and voriconazole by broth microdilution according to the Clinical and Laboratory Standards Institute (CLSI) guidelines (CLSI M27-A3) [ 26 ]. The minimum inhibitory concentration (MIC) values were interpreted according to CLSI document M27-S4 clinical breakpoints [ 27 , 28 ]. The serial concentrations of fluconazole (Sigma-Aldrich, USA) ranged from 0.032 to 64 μg/mL. For itraconazole and voriconazole (Sigma-Aldrich, USA) the serial concentrations ranged from 0.031 to 16 μg/mL.Briefly, the yeast inoculums and suspensions were prepared and counted by the spectrophotometric method at 530 nm. The suspensions were diluted 1:1000 in RPMI 1640 medium (Gibco, UK) with a pH of 7.0 plus 0.165 mol/L MOPS (3-N-morpholinopropane sulfonic acid) and adjusted to a final concentration of 1×10^3^ to 5×10^3^ CFU/mL. Afterward, 100 µL of counted suspension was dispensed into columns 2-11 of 96-well microtiter plates (SPL Life Sciences, Korea) each containing serially diluted antifungal. It should be mentioned that the first column was considered positive control and received no antifungal drug, while column number 12 was considered negative control and received no fungal inoculum. 

The plates were incubated at 35 ºC for 24 h, and MIC values were determined visually based on the concentration at which > 50% inhibition of growth was observed, compared to the growth controls. As recommended by CLSI M27-A3 [ 26 ], the growth inhibition rate of 50% was considered the MIC value. Due to the fact that there were only five Iranian isolates and a total of eight isolates which were less than 10, it was not possible to calculate the MIC50 and MIC90 ranges. However, the geometric mean values were calculated for each tested antifungal.

### DNA Extraction

Genomic DNA was extracted from yeasts via four different methods due to the high sensitivity of the HRM analysis, which needs the lowest concentration of salts in the extracted DNA. These four methods included lithium acetate precipitation [ 29 ], boiling [ 30 ], glass bead beating [ 31 ], and extraction via a commercial kit (Genet Bio, Korea). The concentration and purity of extracted DNA were evaluated by the NanoDrop2000 Spectrophotometer (Thermo-Scientific Inc., USA). The extracted DNA was stored at -20 °C for further molecular assays. 

### Polymerase chain reaction amplification of ERG-11 gene region

Amplification of the *ERG-11* gene was carried out as previously described [ 32 ]. Compatibility of primers CauERG11 F and CauERG11 R with *ERG-11* gene sequences in five Clades (I-V) of *C. auris* was investigated through bioinformatic analysis, and these primers were approved for target gene amplification. The PCR was performed in a 20 µL reaction volume containing 10 µL 2X PCR premix (Ampliqon, Denmark), 5 µM of each forward and reverse Primers and 4 µL of DNA. The PCR started with one cycle of initial denaturation (95 °C for 5 min), 35 cycles of annealing (95 °C for 45 sec, 59 °C for 45 sec, 72 °C for 1 min), and finished with one cycle of final extension (72 °C for 10 min). Extracted DNA from *C. albicans* ATCC 10231 and ultrapure water were included as positive and negative controls, respectively. Finally, the PCR products were analyzed by agarose gel electrophoresis using the Gel Doc XR system (Bio-Rad, USA). 

### Bioinformatic analysis

**Figure 1:**
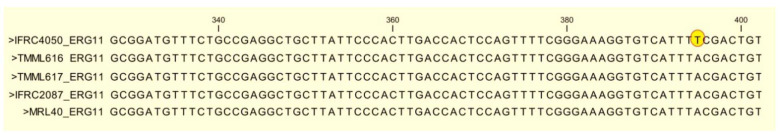
Results of bioinformatic analysis. Suspected Iranian *Candida auris* isolate (IFRC4050) carrying an A-T single nucleotide polymorphism on codon 395 of the *ERG-11* gene

All amplified *ERG-11* targets were sequenced. The results of Sanger sequencing were analyzed and edited via MEGA software (version 5.05). The sequences were checked for similarities with fungal nucleotide sequences submitted to the GeneBank using the Basic Local Alignment Tool Search (BLAST). Moreover, all *C. auris* *ERG-11* nucleotide sequences submitted until February 2022 were obtained from the GeneBank and analyzed via the *CLC* Genomics Workbench software (QIAGEN Inco) to check for the presence of possible SNPs. *Candida auris* standard isolates (CBS10913, CBS12372, and CBS14916) with proven susceptibility and resistance to azoles and without any gene variations in the *ERG-11* region were selected as the wild-type control for alignment analysis to find the mutation of interest (Figure 1).

### Primer design for high-resolution melt curve analysis

Following the in-silico analysis and detection of the 395A>T (Y132F) hotspot mutation, Primer 3 online software was employed for finding specific HRM primer pairs. To achieve the best results, guidelines for HRM primer design was considered. In order to prevent the existence of repetitive elements, the amplicon length was considered to be as short as feasible, between 70 and 150 bp. The optimal criteria for selecting primers included a maximum primer length of 20 bases, a Tm of 58-60 °C, a GC concentration of 30-70%, avoidance of repetitive and consecutively identical oligonucleotides, and absence of more than four consecutive Gs in each primer. Finally, two primer sets, CauERG11HRMF1 (5'-TTCCCACTTGACCACTCCAG-3'), CauERG11HRMR1 (5'-TCTGCTCCATCAACCTCGAG-3') and CauERG11HRMF2 (5'-CGAGGCTGCTTATTCCCACT-3'), CauERG11HRMR2 (5'-TTCTTCTGCTCCATCAACCTC-3') were designed and ordered (Metabion, Germany). A 100 µM solution was prepared and stored at -20 °C and 10 µM working solution was used in each experiment. 

### Real-time polymerase chain reaction and high-resolution melt curve analysis experiments

Real-Time PCR experiments were performed using the HRM-specific dye Eva Green via HOT FIRE Pol Eva Green HRM Mix (Solis Bio Dyne Inc., Estonia). Each PCR-HRM reaction was carried out in a 20 µL total reaction volume, containing 4 µL of the 5X HRM master mix, 0.25 µM of each primer, 2 µL of the DNA template (10-100 ng/µL), and ultra-pure water up to the final reaction volume. Briefly, the thermocycling conditions started with one cycle of initial denaturation at 95 °C for 5 min, 45 cycles with denaturation at 95 ˚C for 15 s and annealing/extension at 60˚C for 45 s followed by the HRM ramping from 60 ˚C to 95 ˚C using QuantStudio Real-Time PCR System (Applied Biosystems, USA). All reactions were performed in duplicate. Furthermore, the specificity of the designed HRM-specific primers was checked through the application of non-auris isolates, including *C. haemulonii*, *C. albicans*, *C. tropicalis*, *C. glabrata*, and *C. parapsilosis* to avoid unspecific annealing of the primers. 

### Test controls

The mutant, wild-type, and control isolates were determined according to the results of their sequence analyses. Three standard *C. auris* strains (CBS10913, CBS12372, and CBS14916) were considered the test control to compare temperature (Tm) and cycle threshold (CT) values with mutant isolate. According to the bioinformatic analyses, the isolate IFRC4050 was considered the mutant, and the rest of them were considered the non-mutant isolates.

### Ethical Considerations

All methods were performed according to the relevant guidelines and regulations. This study was supervised and monitored by the Ethics Committee of Shiraz University of Medical Sciences in 2021 (Permission code: IR.SUMS.REC.1400.874). Informed consent was not applicable to this study.

## Results

### Extracted DNA, antifungal susceptibility testing, and amplification of the ERG-11 gene

Among four different methods of DNA extraction, the glass bead beating method produced the best results of amplification of the *ERG-11* genes, real-time PCR, and HRM analysis experiments due to the lowest salt carryover of DNA and highest yields. Quantitative evaluation of the extracted DNA showed that an average of 17.6 ng/µL of DNA was extracted from each sample. Moreover, the electrophoretic patterns of the *ERG-11* PCRs are presented in Supplementary Figure 1. The results of antifungal susceptibility testing (AFSTs) are presented in Table 1. Briefly, the MIC values for three tested antifungals against isolate IFRC4050 were 64, 0.016, and 1 µg/mL for fluconazole, itraconazole, and voriconazole, respectively. Furthermore, the geometric mean values for three tested antifungals were 12.38, 0.07, and 0.125 for fluconazole, itraconazole, and voriconazole, respectively. 

### Bioinformatic analysis

This study further focused on the presence of the SNPs at the *ERG-11* gene in the resistant isolates. Our *in-silico* analysis indicated that only one resistant isolate, IFRC4050, had an SNP variation on the *ERG-11* gene. A substitution on nucleotide 395 (A to T) leads to an amino acid substitution (Y132F) in this gene. Reaching these results guided us to design Real-time PCR HRM analyses to fast-track the detection of SNP variation on this gene.

### Real-Time polymerase chain reaction and high-resolution melt analyses

**Figure 2:**
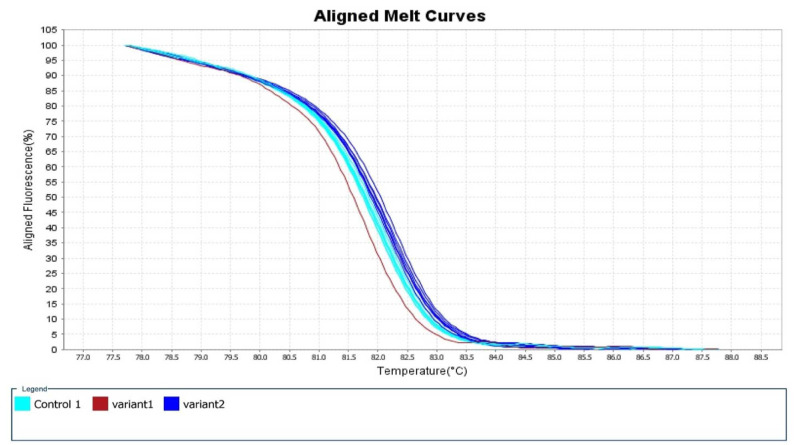
Plot demonstrates the sharp decrease in fluorescence when the double-stranded DNA melts into its single-strand form. Pale blue plot: standard *Candida auris* isolates were considered controls. Blue plot: the rest of the Iranian isolates were considered variant 2. Red plot: the suspected Iranian *C. auris* isolate (IFRC4050) carrying an A-T SNP on codon 395 of the *ERG-11* gene was considered variant 1. Notice: several plots may relate to one isolate due to the test repeat

**Figure 3:**
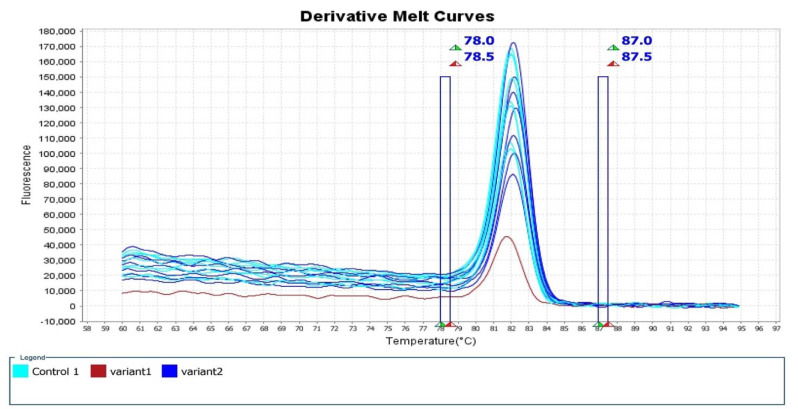
Derivative melt curve plot, which shows the inflection point on the slope as a more easily visualized melt peak. Pale blue plot: standard *Candida auris* isolates were considered controls. Blue plot: the rest of the Iranian isolates were considered variant 2. Red plot: the suspected Iranian *C. auris* isolate (IFRC4050) carrying an A-T SNP on codon 395 of the *ERG-11* gene was considered variant 1. Notice: several plots may relate to one isolate due to the test repeat

**Figure 4:**
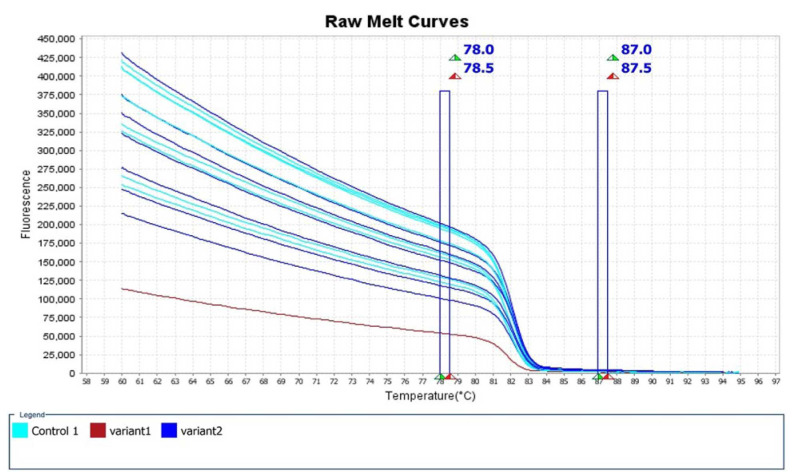
Pale blue plot: standard *Candida auris* isolates were considered controls. Blue plot: the rest of the Iranian isolates were considered variant 2. Red plot: the suspected Iranian *C. auris* isolate (IFRC4050) carrying an A-T SNP on codon 395 of the *ERG-11* gene was considered variant 1. Notice: several plots may relate to one isolate due to the test repeat

**Figure 5:**
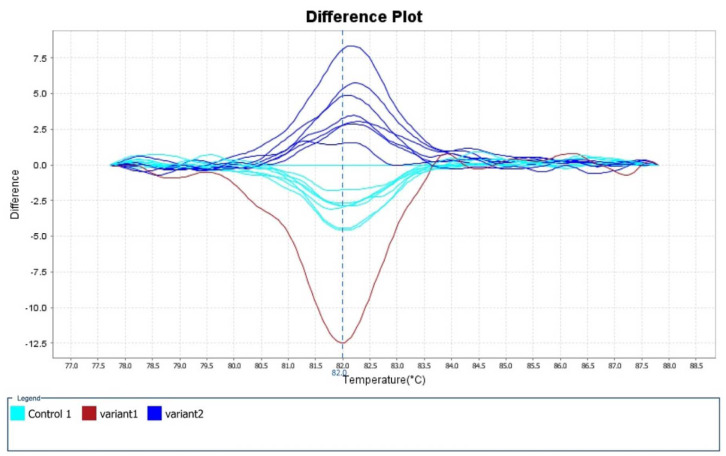
This view accentuates differences between similar melt curves. Pale blue plot: standard *Candida auris* isolates were considered controls. Blue plot: the rest of the Iranian isolates were considered variant 2. Red plot: the suspected Iranian *C. auris* isolate (IFRC4050) carrying an A-T SNP on codon 395 of the *ERG-11* gene was considered variant 1. Notice: several plots may relate to one isolate due to the test repeat

It was found that CauERG11HRM F1R1 primer pairs were superior to CauERG11HRM F2R2 in reaching the optimum CT and Tm values. Therefore, the F1R1 primer set was selected for the main Real-time PCR and HRM analyses. Following the optimization of the reactions, HRM analyses were performed after each Real-time PCR reaction. Table 1 abstracts the mean optimized values and resulting CTs and Tms. The mean Tm range was 81.79-82.39 °C and the mean CT value was 20.06-22.93. Figures 2-5 depict the Real-time PCR and HRM analysis results. 

The standard *C. auris* strains (CBS10913, CBS12372, and CBS14916) (pale blue plots) and the suspected Clade V azole-resistant *C. auris* isolate (IFRC4050) carrying a nonsynonymous A to T SNP on codon 395 of the *ERG-11* gene were considered variant 1 (red plot). Moreover, the rest of the Iranian isolates were considered variant 2 (blue plot) (several plots may relate to one isolate due to the test repeat). 

In Figures 2-5, the melting points were measured by the alteration in fluorescence of the DNA sample. As shown in Figure 2, when the reaction started (at low temperatures), the DNA was double-stranded and the dye was highly fluorescent. In addition, in Figure 3 (derivative melt curves), the Tms are visualized more clearly as peaks. Figure 4 illustrates the pre- and post-melt regions, which are defined by pairs of vertical bars placed before and after the active melt region (78-87.5 °C). The area of data between the pair of bars to the left of the active melt region was used by the software to designate 100% fluorescence, where every amplicon was double-stranded. In Figure 5, the difference between similar melt curves is more clearly visualized. As can be seen in this figure, the target melt curve is melted at the reference region indicated by the vertical plot.

### Test controls

According to bioinformatic analyses, three standard *C. auris* strains (CBS10913, CBS12372, and CBS14916) were considered the test control. The isolate IFRC4050 was considered the mutant and the rest of them were considered the non-mutant isolates. The mean Tm range was 81.79-82.39 °C and the mean CT value range was 20.06-22.93. As indicated in Table 1, regarding the three control isolates, the mean Tm for CT values were as follows: CT = 22.30 and Tm = 81.98 °C for CBS10913, CT = 22.40 and Tm = 81.73 °C for CBS12372, and CT = 21.52 and Tm = 81.81 °C for CBS14916. Besides, a single mutant isolate (IFRC4050) had CT = 20.06 and Tm = 81.79 °C. The CT and Tm values of the rest of the isolates are summarized in Table 1.

## Discussion

*Candida auris* was first reported in Japan where it was isolated from the external ear canal in 2009 [ 33 ]. However, studies have revealed that *C. auris* had already been isolated from South Korea before Japan [ 17 , 34 , 35 ]. In 2011, the first nosocomial fungemia by *C. auris* was reported in South Korea [ 36 ]. During the past several years, *C. auris* has been reported from all inhabited continents and more than 45 countries [ 1 , 2 , 9 ]. A major concern about *C. auris* is the global spread of the yeast and the discovery of new Clades of the yeast that show multidrug resistance to fluconazole and other antifungals. Four Clades of *C. auris* have been approved previously, and finally, the potential fifth Clade (Iranian Clade) was confirmed in 2022. Since no comprehensive study for *C. auris* screening has been conducted in Iran, isolates belonging to this novel Clade have rarely been found. Unfortunately, some of the Clade V isolates are multidrug-resistant, including fluconazole.

Examination of the susceptibility of the clinical isolates to antifungals is another challenging task. Although broth microdilution and other commercial methods for the assessment of the susceptibility of antifungals have been developed, they are not widely used and take a lot of time. Therefore, it is necessary to create an innovative method that can quickly determine antifungal resistance spanning all Clades of *C. auris*. There is no technique that can simultaneously detect every type of antifungal resistance, but the application of different molecular methods can help resolve the problem. Real-Time PCR HRM analysis evaluated in this study demonstrated its ability to identify a mutation that leads to fluconazole resistance in one resistant isolate of *C. auris* belonging to Clade V isolates. In the near future, the development of a platform of HRM assays for the identification of dominant SNPs associated with antifungals may be promising.

 Molecular studies, like STR genotyping and WGS, demonstrated that the fifth Clade formed a separate cluster from the other four major Clades with more than 200,000 SNPs [ 11 ]. Results of antifungal susceptibility testing (Table 1) indicated that the isolates (IFRC4050) were resistant to fluconazole (64 mg/L). Results of a recently published paper by the authors of the present study [ 11 ] indicated that the underlying mechanism of resistance in this isolate was a non-synonymous SNP on nucleotide 395 (A to T) that leads to an amino acid substitution (Y132F) in this gene. According to these results, for the first time in the literature, we developed a double-stranded DNA-binding dye-based HRM assay for rapid detection of the *ERG-11* SNP responsible for fluconazole resistance in *C. auris* isolates. The turnaround time for the experiment, including DNA extraction, is 2 h. We showed that our developed assay was able to successfully cover the results of AFST. These results were covered by the WGS reference method, performed by our colleagues [ 11 ].

Limited data are available about the HRM analysis for mutation screening in *C. auris* until now. However, during the most recent study in 2019, Hou et al. [ 18 ] developed a duplex *ERG-11* assay and a simplex *FKS1* HS1 assay using allele-specific molecular beacons and DNA-melting curve analysis following asymmetric PCR to identify the most prominent resistance-associated mutations in the first four Clade of *C. auris* (Y132F and K143R in *ERG-11*; S639F in *FKS1* *HS1*) within 2 h. Their findings were 100% consistent with DNA sequencing results. The HRM analysis was applied to mutation screening in other species of *Candida*. 

In 2021, Paul et al. [ 37 ] evaluated the tetra primer-amplification refractory mutation system-PCR (T-ARMS-PCR), restriction site mutation, and HRM analysis methods for rapid resistance detection based on the *ERG-11* polymorphism in *C. tropicalis*. They reached acceptable results that can be applied for the rapid detection of *ERG-11* mutations in *C. tropicalis*. In 2016, Caban et al. [ 38 ] proposed an HRM-based system for the *ERG-11* gene for the prediction of azole resistance in *C. albicans* infections. Moreover, the HRM analysis was developed for the rapid identification of *Candida* species by targeting the panfungal *ITS* gene region [ 39 - 43 ].

In this study, the initial tests for confirming the identity of isolates were in concordance with the other previously published papers [ 11 , 22 - 24 ]. Although, in the present study the four DNA extraction methods for HRM assay optimization were tested. The salt carryover directly affected the DNA quality by subtly changing the thermodynamics of the DNA melting transition [ 44 ]. If DNA is not controlled for salt carryover and other experimental artifacts, it will lead to non-specific PCR products, complicated interpretation of DNA melt curves, lower reproducibility, and higher error rates in HRM results of the target isolate (variant) [ 44 - 46 ]. Therefore, it is one of the most effective factors for HRM accuracy. We reached the best and most accurate HRM results when DNA was extracted using the glass bead biting method. The decreased salt and other contaminations resulted in a melt profile that was smoother, more tightly grouped, and easier to separate into clear clusters (Figures 2-5). 

Moreover, we performed two sets of designed primer pairs for post-PCR HRM analyses. These primers were designed according to the recommended guidelines for HRM assay and reagent optimization, as mentioned above. Furthermore, the amplicon was designed for the base change of A/T with a typical Tm curve shift of <0.2 °C and the shortest size (<250 bp). Finally, our results showed that the primer set CauERG11HRM F1R1 reached the best HRM curves and optimum CT and Tm values.

During the last decade, *C. auris* has gradually evolved antifungal resistance, especially to azoles. According to the last announcement by CDC, more than 90% of isolates are resistant to fluconazole while more than 30% of them are resistant to amphotericin B, and 10% of them are resistant to echinocandins [ 6 , 47 - 49 ]. It is estimated that 30-41% and 4% of isolates are multidrug-resistant and pan-resistant to antifungal classes, respectively [ 3 , 14 , 15 , 47 , 50 , 51 ]. Five main molecular mechanisms of azole (fluconazole) resistance were considered for *C. auris* isolates, which were related to the *ERG-11* gene (located at chromosome V).

These mechanisms include I: Point mutations or SNPs in hot spot regions of the *ERG-11* gene that cause amino acid substitutions, *e.g.*, V125A, F126L, F126T, Y132F, K143R, and F444L; *II*: overexpression of the efflux pumps (membrane transporters) of the ATP-binding cassette (ABC) superfamily (CDR1 and CDR2) or major facilitator superfamily (MFS) (MDR1); *III*: functional mutations in *TAC1a* and *TAC1b* and MRR1 (transcription factors that govern ABC and MFS) while the mutations in *TAC1* lead to amino acid substitutions A640V, R495G, F214S, F214L, and S611P; *IV*: overexpression of the *ERG-11* gene; and *V*: chromosome V aneuploidy [ 52 - 55 ]. 

The most common substitutions are found in Clades I and IV (Y132F and K143R). Clade III commonly has an F126L substitution, and Clade II consists of susceptible isolates with rare *ERG-11* mutations [ 3 , 14 , 15 ]. Through sequencing analysis of the *ERG-11* gene, TAC1b D599G and *ERG-11* Y132F mutations were identified in some Clade V of *C. auris* isolates [ 11 ]. Investigation of the *ERG-11* Y132F mutation may help in the early diagnosis of azole resistance as it appears to be a widespread mutation in all *C. auris* Clades. 

The first limitation of the present study was that the developed HRM assay was specific to one of five mechanisms of azole resistance in *Candida* species: a single point mutation of *ERG-11* on nucleotide 395 (A to T) that led to an amino acid substitution (Y132F) in this gene. This mechanism was targeted since it was the most prevalent mechanism of resistance. However, our HRM system may not be applicable to other point mutation detection mechanisms governed by other mechanisms of resistance, as described above. The second limitation was the low number of Clade V isolates in this study. This may be due to the negligence of Iranian physicians and researchers in the diagnosis and identification of infected or colonized patients.

## Conclusion

The prevalence of *C. auris* and its antifungal resistance is a growing issue that worsens the infection situation brought on by the yeast. Therefore, addressing antifungal susceptibility is highly essential. Here, a fast-track HRM-based method was developed to detect one of the most common resistant mechanisms in the *ERG-11* gene, which could make it easier to solve the problem. Our experiments indicated that our developed mutation screening system managed to find the involved mutation in less than 3 h. Nowadays, the application and development of fast-track methods for the detection of resistant isolates will pave the way for controlling the infections caused by multidrug-resistant *C. auris*. However, more studies using various molecular methods are recommended to expand the edges of the field and facilitate the elaboration of rapid and accurate methods of resistance assessment.
